# Supplementary material for: Mercury Exposure and Poor Nutritional Status Reduce Response to Six Expanded Program on Immunization Vaccines in Children: An Observational Cohort Study of Communities Affected by Gold Mining in the Peruvian Amazon
Source: Int J Environ Res Public Health. 2019 Feb 21;16(4):638. doi: 10.3390/ijerph16040638 (PMC6406457; doi:10.3390/ijerph16040638)
Supplement: Supplementary file 1 [file ijerph-16-00638-s001.zip › ijerph-435746-suppl-for xml/ijerph-435746-suppl1-figure_S1.docx]

Supplementary Materials

Calculations to estimate the hair mercury content levels associated adverse neurological endpoints in children used in the analysis.

The 1.2 µg/g hair mercury content level was derived using a blood level associated with adverse neurological impacts in children (5.8 µg/L) and the assumption of a hair:blood ratio of 200:1. The blood level of 5.8 µg/L is recognized by the U.S. Environmental Protection Agency (EPA) and refers to the lower confidence limit of the benchmark dose (BMDL) calculated by the National Research Council. The BMDL refers to a cord blood level associated with adverse child development observed through the Boston Naming Test from the Faroe Islands study. The EPA assumes the ratio of maternal to child blood mercury to be 1:1. A consensus on a hair:blood ratio has not been reached in the literatures and has been observed to range from 140–340 hair:blood (WHO 1990, International Programme on Chemical Safety: Environmental Health Criteria 101, Methylmercury, http://www.inchem.org/documents/ehc/ehc/ehc101.htm).

The 2.0 µg/g hair mercury content level is a level recognized by Peru’s government and the WHO previously. This level has been calculated using a higher benchmark dose and the assumption of a hair:blood ratio of 250:1. The higher benchmark dose refers to the upper range of the BMDL (range: 4.6–7.9 µg/L).

**
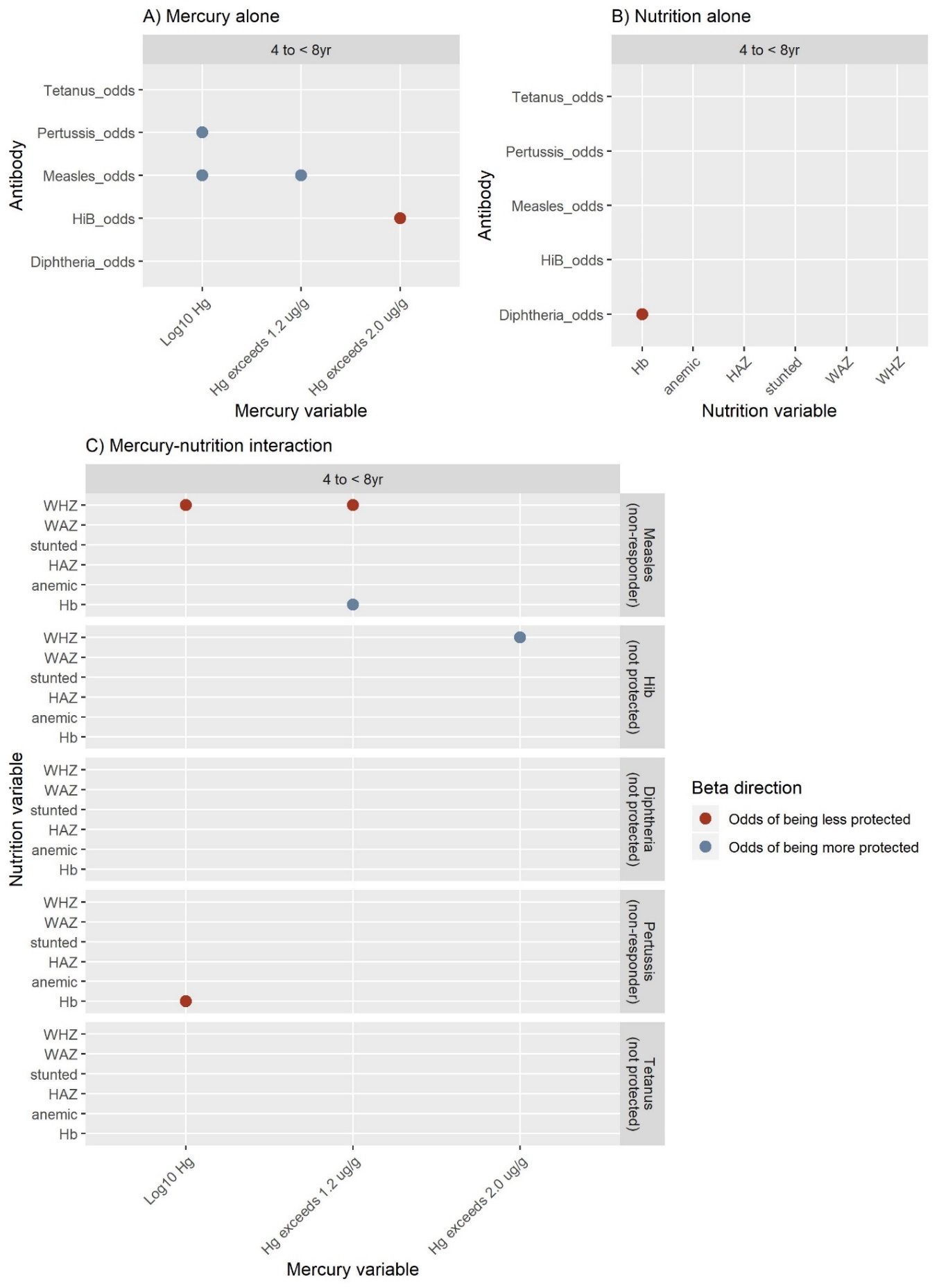
**

**Figure S1.** Summary of model odds ratios for mercury (A), nutritional status (B), and mercury-nutritional status interaction (C) variables in continuous antibody models for older (4 to <8 yr) children. Variables in all models included a mercury variable, a nutritional status variable, the interaction between mercury and the nutritional status variables, sex, and time (time since vaccination for vaccination antibodies or age for total IgG). Hib models for older children were also adjusted for native community. In the mercury alone panel (A) for a specific antibody and mercury variable, a dot signifies that that mercury variable was observed to be significant in at least one model when considered with each nutritional status variable. In the nutritional status alone panel (B) for a specific antibody and nutritional status variable, a dot signifies that that nutritional status variable was observed to be significant in at least one model when considered with each mercury variable. Dot color in A and B panels represents the direction of the average odds ratio for significant models. In the interaction panel (C), a dot signifies that the interaction between the indicated mercury and nutritional status variables was significant. Dot color in the C panel represents the direction of the interaction variable.

**Table S1.** Mean beta estimates (± 95% CI) for variables included in continuous antibody models for younger (<4 yr) and older (4 to <8 yr) children. Model variables included a mercury variable, a nutritional status variable, the interaction between mercury and the nutritional status variables, sex, and time (time since vaccination for vaccination antibodies or age for total IgG). Hib models for older children also included a variable for native community.

- Attached due to size of table

**Table S2.** Odds ratios (± 95% CI) for variables included in continuous antibody models for younger (<4 yr) and older (4 to <8 yr) children. Model variables included a mercury variable, a nutritional status variable, the interaction between mercury and the nutritional status variables, sex, and time (time since vaccination for vaccination antibodies or age for total IgG). Hib models for older children also included a variable for native community. Models were marked as unstable if they did not converge or had a large standard error.

- Attached due to size of table
